# An Uncustomary Branchial Cleft Cyst Presentation With Associated Autoimmune Thyroiditis

**DOI:** 10.7759/cureus.33069

**Published:** 2022-12-28

**Authors:** Meenakshisundaram Senthilnathan, Shivashekar Ganapathy, Bhuvanamha Devi Ramamurthy

**Affiliations:** 1 Department of Pathology, SRM Institute of Science and Technology, Kattankulathur, IND

**Keywords:** branchial arch anomaly, thyroid gland, autoimmune thyroiditis, hashimoto’s thyroiditis, branchial cleft cyst

## Abstract

Branchial cleft cyst is an embryogenic anomaly of branchial apparatus usually occurring in the lateral aspect of the neck. Very few cases of intrathyroidal cystic lesion of branchial cleft have been reported in English literature so far. The patient was a 42-year-old female who presented to the Otorhinolaryngology department with anterior neck mass. The serology revealed elevated antibodies to thyroglobulin and thyroid peroxidase. Fine needle aspiration done in an outside hospital was reported as an epidermal inclusion cyst. Radiology examination of the neck was suggestive of infected fourth branchial cleft cyst. Left hemithyroidectomy was done. On histopathology examination, branchial cleft cyst within the thyroid parenchyma exhibiting features of autoimmune thyroiditis and secondary degenerative changes was noted. This rare case is reported to emphasize multiple-site fine needle aspiration in heterogenous thyroid lesions along with radiological correlation for correct diagnosis and appropriate treatment.

## Introduction

Branchial cleft cysts and other embryogenic anomalies of the branchial apparatus are caused by abnormal involution of the branchial arch [1]. It is typically noticed on the side of the neck and is infrequently found in the oral cavity [2], thyroid [3], thymus, parotid [4], and pancreas [5]. The first branchial cleft cyst accounts for 80% of all cases, followed by the second at 95%, the third at 2%, and the fourth at 1-4% [6]. Louis et al. were the first to report on branchial cleft cysts within the thyroid gland in 1987 [7]. He noted that the cyst wall was made up of many lymphoid follicles with germinal centers and was lined by both squamous and columnar epithelium [7]. Without a final card of clinical differential and radiological correlation, preoperative diagnosis of the lesion is rather difficult. A precise preoperative diagnosis necessitates appropriate management. In this article, we present a rare case of a fourth branchial cleft cyst in a middle-aged female patient with autoimmune thyroiditis.

## Case presentation

A 42-year-old female patient presented with an anterior neck mass that had been steadily progressing for eight months. The results of the thyroid function test were normal (thyroid-stimulating hormone [TSH] = 0.08 µIU/ml, Free T3 = 3.99 pg/ml, Free T4 = 1.72 ng/dl). Thyroglobulin antibody levels increased (310 IU/ml) as well as thyroid peroxidase antibody levels (124 IU/ml). A fine needle aspiration performed outside of the hospital revealed an epidermal inclusion cyst with mature and anucleate squamous cells.

**Figure 1:**
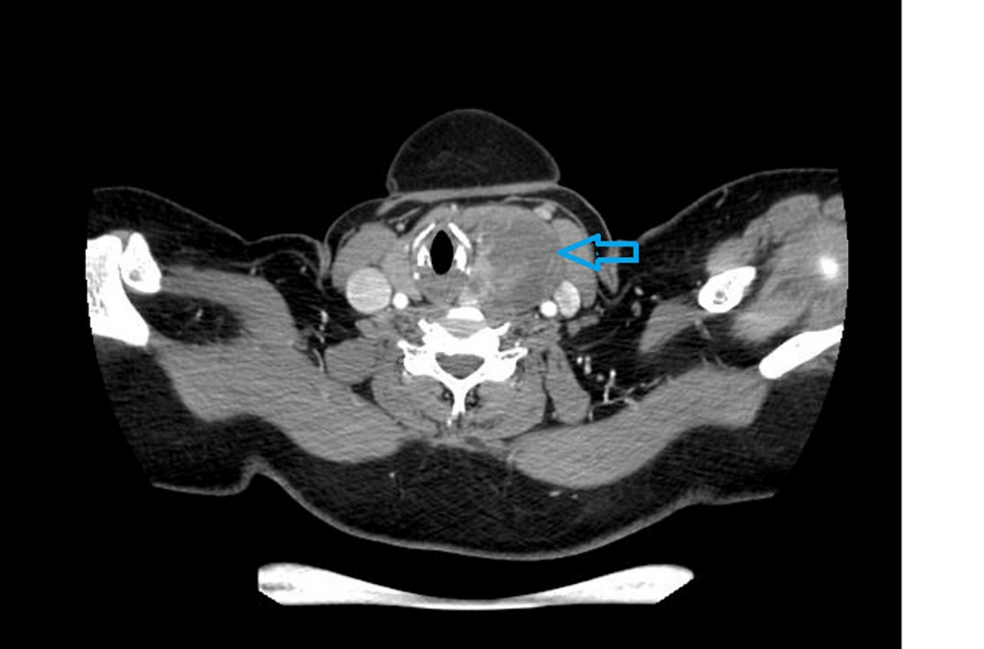
CT neck showing a cystic lesion of size measuring 5.2x4.5x3.4cm abutting the left lobe of thyroid with internal septations.

A cystic lesion measuring approximately 5.2x4.5x3.4 cm with its epicenter in the left pyriform sinus, extending inferiorly up to the left lobe of the thyroid, and containing a few enhancing internal septations was discovered by CT scan of the neck (Figure 1), suggesting an infected fourth branchial cleft cyst. The right thyroid lobe appeared to be normal with no cervical lymphadenopathies in the CT scan. A left hemithyroidectomy was performed on the patient.

**Figure 2:**
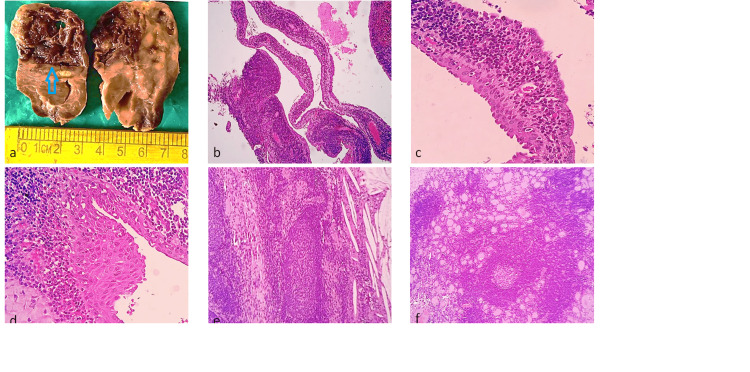
(a) Macroscopic image of left hemithyroidectomy specimen. (b) Cyst wall with abundant lymphoid aggregates (10x). (c, d) Cyst wall lined by pseudostratified columnar epithelium and stratified squamous epithelium with dense lymphoid tissue (40x). (e) Cholesterol clefts, foamy macrophages, lymphoid follicles, and compressed thyroid parenchyma (10x). (f) Adjacent thyroid parenchyma showing atretic thyroid follicles and lymphoid follicles (10x).

Grossly, left hemithyroidectomy specimen was capsulated and measured 6.5x4x3 cm. The cut surface revealed a grey-brown discolored cystic area approximately 3.5x2.5 cm in the upper pole, with internal septations. The surrounding area was discolored from grey-white to grey-brown, with focal yellow spots (Figure 2A). 

A cyst wall lined by stratified squamous epithelium and in a few locations bi-layered columnar epithelium were observed under a microscope within the remnant thyroid parenchyma. It has lymphoid follicles and extensive lymphocytic infiltration in its fibrous tissue wall. Atretic thyroid follicles, foamy macrophages, cholesterol clefts, and lymphoplasmacytic infiltrates were seen (Figure 2B-2F). The definitive diagnosis of intrathyroidal branchial cleft cyst with autoimmune thyroiditis was given.

## Discussion

So far, only around 33 cases of intrathyroidal branchial cleft cyst have been documented in the literature, and only very seldom from India [3,7-10]. The age range spanned from the first to the eighth decade (average age = 45.6 years), with a female preponderance (21 cases) like the current case [3,7-10]. Similar to the present case, authors have noted that branchial cysts were discovered to affect the left lobe of the thyroid more frequently (13 cases) than the right lobe [3,7-10]. A small number of cases involving both lobes were also reported [7-10]. Age, gender, and the laterality of the lesion have all been specifically implicated, but their significance has not yet been determined [8].

There is still debate concerning the pathophysiology of the branchial cleft cystic lesion within the thyroid gland. However, there are two theories that have been put out, one of which states that the ultimobranchial rests, which are solid cell nests, undergo cystic degeneration [8,9]. Squamous metaplasia of the cystic degeneration of the nodular goiter or tumor with secondary chronic inflammation is the second notion that has been put forth [8,9].

The majority of the cases reported were associated with thyroid lesions such as the current case with Hashimoto's thyroiditis (10 cases), chronic lymphocytic thyroiditis (six cases), papillary thyroid cancer (three cases), nodular hyperplasia (two cases), and multinodular goiter (one case) [3,7-10]. Chronic lymphocytic thyroiditis with papillary carcinoma was found in three cases [3]. Hashimoto's thyroiditis with papillary carcinoma was found in one case, and multinodular goiter was found in one case together with chronic lymphocytic thyroiditis [7-10]. Additionally noted were heterotrophic parathyroid [3,8], thymus [3,8], and salivary glands [8]. No thyroid lesion was present in four cases [3,7-10].

It is important to distinguish between intrathyroidal branchial cleft cyst and other thyroid lesions with squamous epithelium, such as congenital remnants, benign metaplasia, and malignancy [8]. Thyroglossal duct cysts, cystic thymic remnants, and epidermal inclusion cysts are examples of congenital remnants. Goiter and thyroiditis can both show signs of benign metaplasia. Papillary carcinoma, mucoepidermoid carcinoma, squamous carcinoma, adeno-squamous carcinoma, and teratoma are thyroid tumors that can display squamous metaplasia. There was no evidence of congenital remnants, benign metaplasia, or neoplasm in the current case.

In the epithelial lining of a branchial cleft cyst, a few authors have found positive immunohistochemistry (IHC) staining with galectin 3, high molecular weight cytokeratin (HMWCK), and localized staining with carcinoembryonic antigen (CEA) [9]. Thyroglobulin and calcitonin are two negative immunohistochemistry stains [9]. But in this case, it wasn't done, as studies have shown IHC was non-significant and the diagnosis was made with clinical, radiological and histopathological findings.

## Conclusions

This case is being described since it is unusual and to highlight the importance of performing multiple site fine needle aspiration in heterogenous thyroid lesions for accurate diagnosis. Cytological suspicion of cystic lesions with squamous epithelial cells or columnar epithelial cells can lead to the preliminary preoperative diagnosis of a branchial cleft cyst. The puncture biopsy with a fine needle is not always reliable, as it is difficult in the event of a cystic lesion of the thyroid and is subjective by the pathologists. It requires trained pathologists accustomed to these pathological situations. Alternatives to fine needle biopsy puncture like frozen section examination can be done. Despite its rarity, the likelihood of recurrence and malignant transformation should not be underestimated. Therefore radiological correlation is crucial for a conclusive diagnosis and preparation for the right surgical therapy.
